# Temperature-Induced Change of Water Structure in Aqueous Solutions of Some Kosmotropic and Chaotropic Salts

**DOI:** 10.3390/ijms222312896

**Published:** 2021-11-29

**Authors:** Ferenc Kovács, Hui Yan, Heng Li, Sándor Kunsági-Máté

**Affiliations:** 1 Institute of Organic and Medicinal Chemistry, Faculty of Pharmacy, University of Pécs, Honvéd útja 1, H-7624 Pécs, Hungary; kovacsf@gamma.ttk.pte.hu; 2 Department of Physical Chemistry and Materials Science, Faculty of Sciences, University of Pécs, Ifjúság útja 6, H-7624 Pécs, Hungary; 3 Tianjin Key Laboratory of Photoelectric Materials and Devices, School of Materials Science and Engineering, Tianjin University of Technology, Tianjin 300384, China; yanhui@tjut.edu.cn; 4 Key Laboratory of Display Materials and Photoelectric Devices, Tianjin University of Technology, Ministry of Education, Tianjin 300384, China; 5 Fujian Provincial Key Laboratory of Semiconductors and Applications, Collaborative Innovation Center for Optoelectronic Semiconductors and Efficient Devices, Department of Physics, Xiamen University, Xiamen 361005, China; liheng3000@xmu.edu.cn; 6 Jiujiang Research Institute, Xiamen University, Jiujiang 332000, China; 7 János Szentágothai Research Center, Ifjúság útja 20, H-7624 Pécs, Hungary

**Keywords:** water structure, hydrogen bond, water cluster, Raman spectroscopy

## Abstract

The hydrogen bond structure of water was examined by comparing the temperature dependent OH-stretching bands of water and aqueous NaClO_4_, KClO_4_, Na_2_SO_4_, and K_2_SO_4_ solutions. Results called attention to the role of cations on top of the importance of anions determining the emerging structure of a multi-layered system consisting single water rings or multi-ring water-clusters.

## 1. Introduction

The main medium along the metabolic pathways, pharmaceutical and biological processes is water. The characteristic structure of water in condensed, solid and liquid, phases is determined by the existing hydrogen bond system between water molecules. Water is a protic solvent, and therefore plays an extraordinarily important role in life. In aqueous solutions there is a competition between weak interactions of individual water molecules and interactions between water molecules and solutes [1,2,3]. Differently sized and structured liquid phase water clusters are in competitive equilibrium with each other because of the few ten kJ/mol bonding strength of hydrogen bonds. Due to the weak behavior of both the water-water and the water-solute interactions, temperature and dissolved solutes can fundamentally affect the structure of water in a liquid phase.

Solutes are characterized as kosmotropic or chaotropic when they improve or destroy the order of water structure by stabilizing or disrupting water-water interactions. Small size and high charge density accompany kosmotropic ions and large sizes with low charge density chaotropes [4].

Particular interest surrounds the weak interactions of water molecules and biomolecules, even though the structure of water itself is a very interesting topic in its own right. Our research group conducted several investigations through the last 20 years on the field of weak interactions between biologically important molecules in aqueous media with a focus on how water molecules reorganize themselves after leaving the hydration shell of the molecules interacted [5,6]. Besides the structure of pure water, the weak interactions of water and bioactive molecules attract distinct attention in the fields of pharmacy, life, and medical sciences also through quantum chemical topology [7,8] because the equilibrium of complex formation is fundamentally dependent on the variation of entropy.

It is well-known, that the wide band around 3200 cm^−1^ and 3400 cm^−1^ in the Raman spectra of water is associated with molecular vibrations coming from the stretching of O-H···O bonds and a Raman spectroscopy commonly used to investigate water structure. There is still no consensus on how to deconvolute the aforementioned wide band [9,10], it is a challenge to identify the particular characteristic vibrations: the same enveloping curve can be made of differently weighted and different number of peaks; therefore, different research groups have quite different results. Plenty of experimental and theoretical work is carried out to describe the structure of bulk [11], liquid [12,13,14,15,16], supercritical [13] water molecules confined in different media [17,18], on different surfaces [19,20] and even in the effect of different isotopes [21]. Several models were built to describe linear or cyclic (H_2_O)_n_ clusters of different sizes (n = 2, 3, 4, …, 60) and spatial clusters either fullerene-like macro (n = 100, 280, 320) structures [22,23] or formed of those cyclic clusters. Previously performed experimental and modelling works showed the temperature dependence of the size and distribution of water clusters and clusters of primary alcohols [24,25,26,27].

This study focuses on the change of water structure in lieu of the ionic solutes of chaotropic sodium and potassium perchlorate and the kosmotropic sodium and potassium sulphate. The samples were excited by non-coherent radiation of Xe-lamp and the temperature-dependent Raman spectra were recorded.

## 2. Results

**Figure 1:**
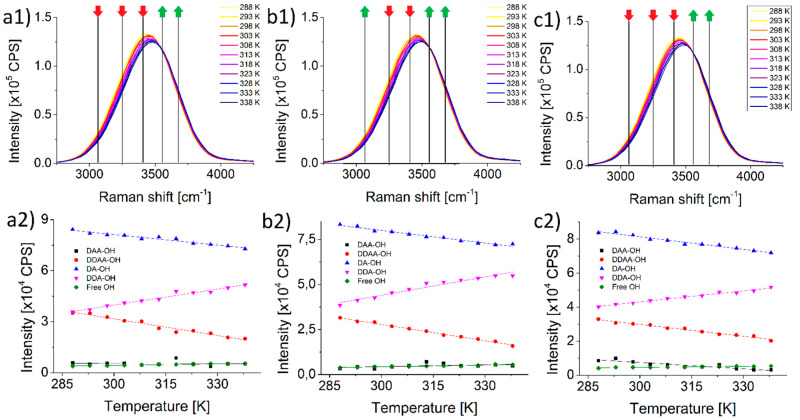
Temperature dependent Raman signals of (**a1**) pure water, (**b1**) 100 mM aqueous KClO_4_ and (**c1**) 100 mM aqueous NaClO_4_ solutions. Changes of the amplitudes of Gaussians used to superpose the Raman spectra plotted against temperature (**a2**,**b2**,**c2**). Red and green arrows indicate the decrease and increase in the amplitude of the respective deconvoluted Gaussians caused by increasing temperature.

Figure 1(a1–c1) shows the Raman spectra of pure water, potassium and sodium perchlorate solutions, respectively. The three series are nearly identical, no considerable difference can be observed. Figure 1(a2–c2) insets show a more refined picture. Even though the amplitude changes look similar after deconvolution, Table 1 details how differently the two salts behave. In the case of peaks DDAA-OH and DA-OH the change is clearly growing with the cation compared with pure water. On the other hand, with the three other species (DAA-OH, DDAA-OH and free OH) the two salts behave quite differently, opposing each other. The number of water molecules engaged in the DAA-OH bond grow significantly in the presence of potassium perchlorate, and drops rapidly in the presence of sodium perchlorate compared with pure water. In the case of DDA-bonded and free water molecules the changes are more pronounced and stronger in the potassium perchlorate solution, while in the sodium perchlorate solution they are milder than in pure water.

**Figure 2:**
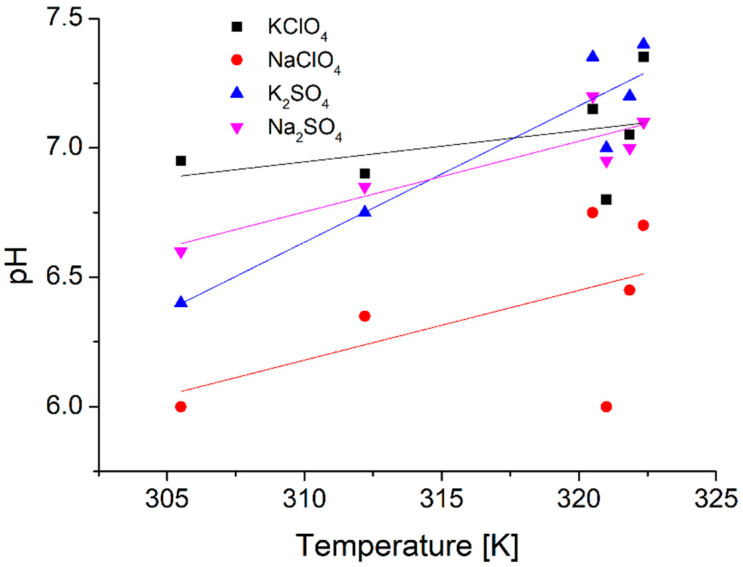
Temperature dependent pH measurements of 100 mM aqueous solutions of KClO_4_, NaClO_4_, K_2_SO_4_, and Na_2_SO_4_.

Measurements also showed another difference between pure water and aqueous perchlorate solutions, the pH of water dropped (−0.64), while those of the salt solutions rose (+0.89 for potassium and +1.43 for sodium perchlorate) to a 50 K change in temperature (Figure 2).

**Figure 3:**
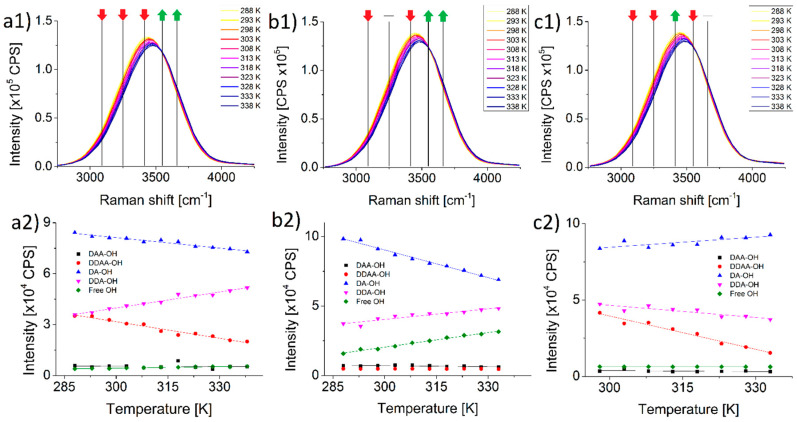
Temperature dependent Raman signals of (**a1**) pure water, (**b1**) 100 mM aqueous K_2_SO_4_ and (**c1**) 100 mM aqueous Na_2_SO_4_ solutions. Changes of the amplitudes of Gaussians used to superpose the Raman spectra plotted against temperature (**a2**,**b2**,**c2**). Red and green arrows indicate the decrease and increase in the amplitude of the respective deconvoluted Gaussians caused by increasing temperature, a dash indicate no significant change in these.

On Figure 3(a1–c1) insets it is shown how the Raman spectra of 100 mM aqueous K_2_SO_4_ and Na_2_SO_4_ solutions differ from that of ultrapure water. The three spectra are virtually identical.

On Figure 3(a2–c2) insets it is shown how the course of both of DA-OH and DDA-OH peaks are the complete opposite of each other in the case of these two salts. The number of DA-bonded water molecules in pure water drops with rising temperature (even more pronounced in K_2_SO_4_ solution) rises in Na_2_SO_4_. The same plays out with DDA-bonded water molecules, in pure water and in K_2_SO_4_ solution the peak rises, while in Na_2_SO_4_ solution it drops significantly. The number of DDAA-bonded water molecules interestingly stays the same in the K_2_SO_4_ solution but drops in Na_2_SO_4_ solution, the opposite is true for the number of free water molecules.

Measurements showed an even greater rise of pH for the sulfate solutions: +2.84 for the potassium and +1.31 for the sodium sulfate solvents for the 50 K temperature change.

It was recently confirmed that the lifetime of water clusters in liquid water is extremely short, it is less than a few picoseconds [28]. Within this timeframe the hydrogen bonds continuously form and disappear emphasizing the weak interactions between water molecules. It is a daunting challenge to design an appropriate model based on such weak interactions, especially if we consider that the molecular environment itself has a deep impact on these interactions. Although there are several solvent models to take the environment into account in this particular case, the cluster is surrounded by other clusters, whose structure is also unknown. To take at least a small step towards the true picture of reality we decided to calculate the stability of (H_2_O)_3_ clusters as a function of temperature in the absence and presence of potassium and sodium ions. Table 2 summarizes these results.

Compared with our previous study of (H_2_O)_3_ clusters in a vacuum [29] a lower stability is obtained after the introduction of an aqueous environment through the PCM method. The stability of clusters decreases at higher temperatures. Even though the presence of potassium and sodium ions slightly weakens the cluster stability, they have an opposite temperature dependent effect, the presence of potassium ions decreases, and the presence of sodium ions increases the cluster stability. This result is in agreement with our experiments. It can be clearly seen that the formation of (H_2_O)_3_ clusters is enthalpy-driven in the case of pure water and entropy-driven in the presence of sodium ions. In the case of potassium ions, the entropy change is also negative, but it is a much smaller number than it is observed for pure water. It must be noted however that even though this model justifies our experimental results there can also be other underlying processes in the works in the solution supplying a congruent effect. Further examinations are necessary to clarify the processes at the molecular level.

## 3. Discussion

**Figure 4:**
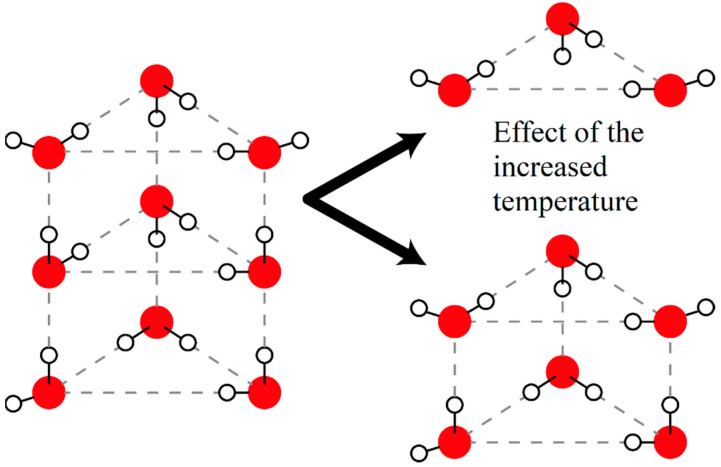
A schematic model of (H_2_O)_9_ clusters breaking off to (H_2_O)_3_ rings and (H_2_O)_6_ clusters [30] due to the higher temperature.

In the aqueous KClO_4_ solution it was observed that the number of DAA- and DDA-bonded water molecules rises and the number of DDAA-bonded water molecules drops with increasing temperature. This phenomenon may be caused by the breaking of multi-ring clusters into smaller pieces (see Figure 4). The water molecules sitting in the middle ring are DDAA-bonded until the moment the top ring breaks off this cluster, when this happens those water molecules will be bonded only with three other water molecules and so their state becomes DAA- or DDA-bonded instead of the previous state. The water molecules constituting the top ring are DAA- and DDA-bonded up until the break off, then they become bonded only by themselves forming a ring with DA-bonds. The measurable increase in the pH is probably caused by the reversal of the usual temperature-induced dissociation of water molecules.

In the aqueous K_2_SO_4_ solution, the number of DDAA-bonded water molecules (and so multi-ring clusters) is practically preserved meanwhile the DA-bonded peak loses and the free water peak gains significance dramatically. This may mean that in the K_2_SO_4_ solution, the primary source of free water molecules in temperature-induced breakdowns, are DA-bonded water molecules or water rings. At the same time the multi-ring clusters seem to be preserved from the same breaking up. The pH change was by far the most pronounced of all the samples in the presence of K_2_SO_4_, which concurs with the most significant rise in the number of free water molecules. This is possibly due to the association of hydrogen and oxonium ions.

In the aqueous Na_2_SO_4_ solution the opposite seems to happen, the number of DAA-, DDAA- and DDA-bonded water molecules drop (the DDAA-OH dramatically) while the number of DA-bonded molecules rises significantly, and the free ones stay the same. This suggests that multi-ring clusters break off into single rings, but those rings become more and more stable and their presence pronounced. It is interesting that the number of free water molecules is constant even if the pH rises. It is a fairly obvious assumption that the previously dissociated water molecules recombine at the same rate as previously free water molecules form up into rings, all the while the temperature increases.

**Figure 5:**
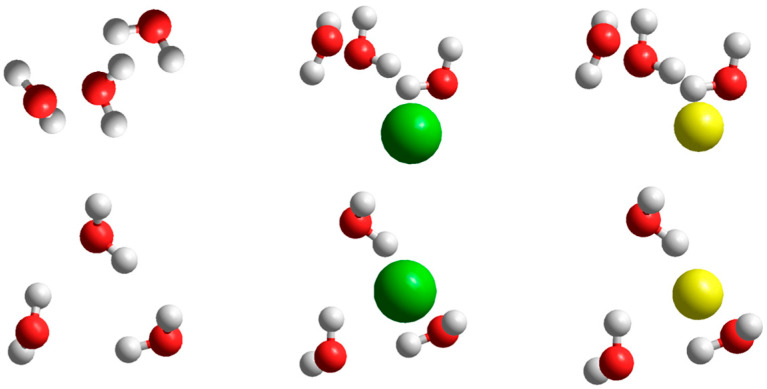
Side (top row) and top (bottom row) of the optimized structures of (H_2_O)_9_ clusters (**left**), (H_2_O)_9_ clusters with potassium ion (**middle**) and (H_2_O)_9_ clusters with sodium ion (**right**).

A quantum-chemical investigation confirms the presence of stable (H_2_O)_9_ clusters. Optimized structures in the absence or in the presence of potassium and sodium ions are shown in Figure 5. The structure of (H_2_O)_9_ clusters are very similar to those proposed by Tachikawa [31], and the presence of potassium or sodium ions does not change the structure significantly.

## 4. Materials and Methods

### 4.1. Chemicals

A Merck Elix Essential water purification system combined with a Progard TS2 Pretreatment Pack and a Type 2 Water vent filter was utilized to produce ultrapure water. The conductivity (less than 0.1 µS/cm) check made sure that the expected water quality is achieved. Spectroscopic grade sodium perchlorate, and potassium perchlorate, and sodium and potassium sulfate were obtained from Reanal Budapest and was used as received. A concentration of 100 mM aqueous solutions of NaClO_4_, KClO_4_, Na_2_SO_4_, and K_2_SO_4_ were prepared using pure water as a solvent.

The two-point calibration of the pH meter was performed using the Scharlau buffer solutions as received. The Scharlau potassium hydrogen phthalate buffer solution acted as a pH 4 and the Scharlau sodium carbonate/sodium hydrogen carbonate buffer acted as pH 10 calibration solution.

### 4.2. Instruments and Experimental Setup

For measurements, a Jobin Yvon—Spex Fluorolog tau 3 spectrofluorimeter with a Xenon light source was used. The Raman scattering of water was recorded within the 365 nm–450 nm spectral range by 0.5 nm steps using 0.5 s integration time. Both for the excitation and emission sides a bandwidth of 3 nm was used. Each spectrum was constructed by averaging 50 Raman spectra measured from 288 K to 338 K in 5 K increments. For data evaluation OriginPro 8.5.1 was used.

For pH-measurements a two-point calibrated Adwa AD2000 pH and temperature meter was used.

### 4.3. Theoretical Calculations

The water cluster structures were optimized using the B3LYP density functional [32,33] augmented with the D3 dispersion correction [34] of Grimme et al. The geometry optimizations and the vibrational analysis were performed using the TZVP basis set [35]. The intra-cluster interaction energies (*E_int_*) were determined according to Equation (1) using the QZVP basis set [36]. Energies are corrected for the basis set superposition error (BSSE) [37]:

(1) $$E_{inter}=E_{cluster}-3\cdot E_{H_{2}O}\;or\;E_{inter}=E_{cluster+ion}-3\cdot E_{H_{2}O}-E_{ion}$$

where *E* is the total energy of the species interacted. Gibbs free energies of interaction Δ*G_int_* were obtained from the BSSE-corrected B3LYP-D3/QZP interaction energies combined with non-thermal (ZPE) and thermal corrections *dG_corr_* resulting from the TZVP frequency analysis at *T* = 298.15 K and *p* = 1 atm. The temperature-dependence of the entropy is considered through the following equation:

(2) $$S_{vib}=R\underset{i}{\sum }\left\{\frac{h\nu_{i}/kT}{e^{\left(h\nu_{i}/kT\right)-1}}-ln\left[1-e^{\left(-h\nu_{i}/kT\right)}\right]\right\}$$

where *ν_i_* is the frequency of vibration and *T* is the temperature.

For the rotational partition function no symmetry is considered; therefore, the appropriate symmetry number *s* = 1 is chosen. Gibbs free energy changes are calculated as follows:

(3) $$\Delta G_{int}=E_{int}\left(BSSE-QZVP\right)+\left[dG_{cluster}^{corr}-\sum dG_{monomers}^{corr}\right]$$

All calculations were performed with a very tight SCF cutoff and integration grid size of 5, together with tight geometry optimization criteria. In addition to B3LYP-D3 the M06-2X density functional [38,39] as implemented in the Gaussian 09 program [40] with the aug-cc pVTZ basis set [41] was also used. The PCM solvent model implemented in the Gaussian code is applied to consider the high permittivity of water [42].

### 4.4. Data Processing

**Figure 6:**
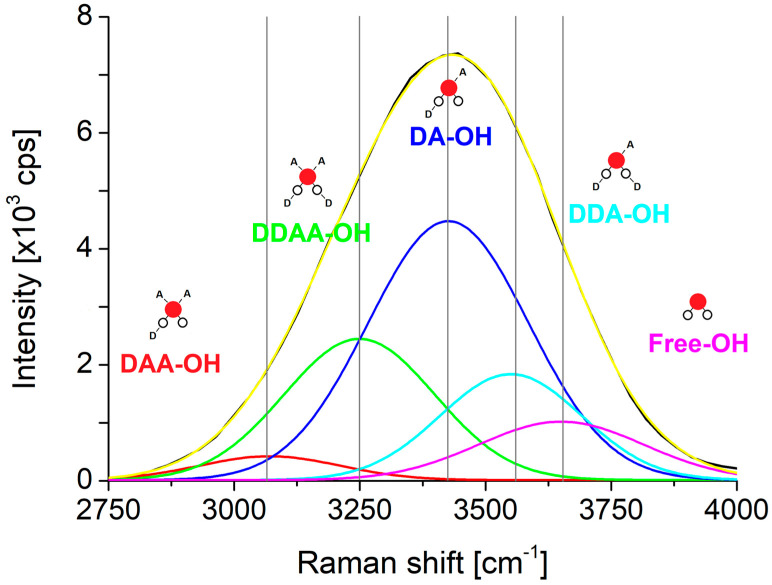
Structure of the typical Raman spectrum in the OH-stretching region of water molecules acting on different levels of proton-acceptors and proton-donors. D—hydrogen donor, A—hydrogen acceptor [26,43].

The OriginPro 8.5.1 custom fitting function was used to deconvolute the respective Raman signals of the fluorimeter (Figure 6), which is the sum of five Gaussian peaks:

(4) $$y=y_{0}+\overset{5}{\underset{i=1}{\sum }}A_{i}\cdot e^{-\frac{{\left(x-x_{c_{i}}\right)}^{2}}{2\omega_{i}^{2}}}$$

The software took a global search for the shared values of the five Gaussians’ peak position throughout the measured spectra. The base, amplitudes and widths were chosen as variable parameters for fitting.

Due to the fact that the area of the peaks does not specify the exact quantity of the respective species the deconvolution itself is not a definite method. The same cumulative curve can be the result of many different sets and variations of the Gaussians with the same exact location. One accepted solution came from the identification of a different bonding scheme of water molecules, depending on the role of a given water molecule as proton donor or proton acceptor in the formation of hydrogen bonds the associated vibrations can be used to design an appropriate set of Gaussians. A water molecule theoretically can form DDAA, DDA, DAA, DA, DD, AA, D, or A types of local hydrogen bonding [43]. DD and AA structures are disfavored, having slightly repulsive two-body components, D and A can be reasonably ignored at ambient pressure according to the Boltzmann distribution [43]. Therefore, at ambient temperature the main local hydrogen bonding can be expected to be DDAA, DDA, DAA and DA [43]. The fifth Gaussian comes from the OH-vibration of free water molecules. If the resulting Gaussians are proportional to those species that are linked to them, is not known. The change of the area of these deconvoluted Gaussians is proportional to the change of the amount of the respective species can be stated.

Ph measurements took place between 303 K and 323 K, the values for the whole 50 K range were linearly extrapolated.

## 5. Conclusions

In this study a deconvolution of temperature-dependent Raman spectra was performed in order to gain some insight about the structure of water via the change of differently bonded water molecule species. Ultrapure water and aqueous solutions of KClO_4_, NaClO_4_, K_2_SO_4_, and Na_2_SO_4_ were investigated to determine the chosmotropic and kaotropic effect of the different ions in this particular view.

In the KClO_4_ solution the multi ring water clusters formed of three (or more) rings seem to break up into smaller double and single-ring clusters. In the aqueous K_2_SO_4_ solution single water rings seem to break up into free water molecules. Meanwhile the more complex three (or more)-ring clusters seem to be stabilized and preserved in numbers. Interestingly in the Na_2_SO_4_ solution the same complex water clusters break up into single rings but in turn these rings seem to be stabilized and preserved in number.

The topological surface area and electric charge difference explains why sulfate ions have a much stronger effect on neighboring water clusters than perchlorate ions. In the latter case water rings are much more easily broken up into free water molecules with the increasing temperature.

Sodium and potassium ions are neighbors in the Hofmeister series, and it is generally accepted that usually they do not contribute much to the structure of water. A potassium ion has a roughly 1,5-fold larger surface area than a sodium ion, which causes the electronegativity of sodium to be higher that of potassium. This higher electronegativity can potentially attract and turn the lone pairs of oxygen in water to themselves bringing about the water rings into a spherical shell. Larger, multi-ring clusters obviously do not fit well into a thin shell such as this, while single water rings oriented tangential to each other can easily constitute another layer leading to a buildup of a larger, more complex system in a layered fashion. It is known that the different bonding state of a water molecule affects the charge transfer, bond length, and strength not only all of its own bonds, but all the bonds of the water molecules bonded to that single water molecule [44,45,46,47]. This cooperative strengthening/weakening effect of the water cluster system ripples through at least the nearby vicinity changing the already existing clusters into energetically favorable compositions. The same can happen around potassium ions forming thicker layers, consisting multi-ring clusters.

Another interesting aspect of these salt solutions was the observed rise in the pH by temperature. This phenomenon is likely the result of the association of hydrogen and oxonium ions into the otherwise dissociated water molecules. The presence of chaotropic perchlorate ions shows this to a lesser degree (+0.89 and +1.43), meanwhile the presence of kosmotropic sulfate ions shows this to a larger extent (+2.84 and +1.31). The presence of potassium cations was more extreme (+0.89 and +2.84), and the presence of sodium cations showed a more uniform (+1.43 and +1.31) change, even though the respective ionic strengths are different in the perchlorate and sulfate solutions.

Although the modelling results are in agreement with the experimental results, further investigation is necessary to accurately describe the system at the molecular level since the PCM method cannot take the clustered environment into account. Considering the cluster formation around the clusters investigated in this study the relative permittivity of the solution around the selected clusters likely differs from the relative permittivity of the bulk. 

## Figures and Tables

**Table 1:** Slopes of lines fitted to the amplitudes of the Gaussians from the deconvolution of the Raman spectra of water and pH change, 100 mM aqueous solutions of KClO_4_, NaClO_4_, K_2_SO_4_, and Na_2_SO_4_.

	DAA-OH	DDAA-OH	DA-OH	DDA-OH	Free OH	pH
Pure water	−6	−325	−205	314	29	−0.64
100 mM KClO_4_	35	−301	−233	339	32	+0.89
100 mM NaClO_4_	−123	−231	−246	216	21	+1.43
100 mM K_2_SO_4_	−22	−1	−664	255	350	+2.84
100 mM Na_2_SO_4_	−20	−728	221	−265	−1	+1.31

**Table 2:** Gibbs free energy, enthalpy (in kJ/mol), and entropy (J/K∙mol) changes associated to the formation of (H_2_O)_3_ clusters.

	Type of Calculation	B3LYP-D3/QZVP			M06-2X/aug-cc-pVTZ		
	Temperature (K)	ΔG	ΔH	ΔS	ΔG	ΔH	ΔS
Pure water	280	−28.59	−51.7	−82.54	−28.47	−51.3	−81.57
	290	−28.18	−51.7	−81.12	−27.68	−51.3	−81.48
	300	−27.43	−51.7	−80.93	−26.89	−51.3	−81.37
	310	−26.75	−51.7	−80.51	−26.11	−51.3	−81.29
	320	−26.06	−51.7	−80.14	−25.46	−51.3	−80.78
	340	−24.53	−51.7	−79.92	−23.86	−51.3	−80.71
Water + potassium	280	−20.05	−23.5	−12.33	−19.17	−22.9	−13.34
	290	−19.94	−23.5	−12.29	−19.05	−22.9	−13.28
	300	−19.83	−23.5	−12.26	−18.94	−22.9	−13.22
	310	−19.72	−23.5	−12.21	−18.82	−22.9	−13.18
	320	−19.61	−23.5	−12.18	−18.7	−22.9	−13.14
	340	−19.37	−23.5	−12.15	−18.45	−22.9	−13.11
Water + sodium	280	−26.09	−13.4	45.31	−25.27	−12.9	44.15
	290	−26.52	−13.4	45.23	−25.69	−12.9	44.07
	300	−26.94	−13.4	45.11	−26.09	−12.9	43.96
	310	−27.36	−13.4	45.01	−26.51	−12.9	43.88
	320	−27.80	−13.4	44.98	−26.94	−12.9	43.85
	340	−28.68	−13.4	44.94	−31.17	−12.9	53.73

## Data Availability

The data presented in this study are available on request from the corresponding author.
